# Clinical evaluation of resection of functional area gliomas guided by intraoperative 3.0 T MRI combined with functional MRI navigation

**DOI:** 10.1186/s12893-024-02506-z

**Published:** 2024-07-27

**Authors:** Luoyi Tian, Nan Peng, Zhongrun Qian, Jinpeng Hu, Wei Cheng, Yanghua Xia, Chuandong Cheng, Ying Ji

**Affiliations:** 1 Department of Neurosurgery, The First Affiliated Hospital of USTC, Division of Life Sciences and Medicine, University of Science and Technology of China, No. 1, Swan lake road, Shushan district, 230001 Hefei, Anhui China; 2 Department of Neurosurgery, The Affiliated Provincial Hospital of Anhui Medical University, No. 1, Swan lake road, Shushan district, 230001 Hefei, Anhui China

**Keywords:** Functional area, Glioma, Intraoperative magnetic resonance imaging, Multimodal functional magnetic resonance imaging, Neuronavigation

## Abstract

**Background:**

In assessing the clinical utility and safety of 3.0 T intraoperative magnetic resonance imaging (iMRI) combined with multimodality functional MRI (fMRI) guidance in the resection of functional area gliomas, we conducted a study.

**Method:**

Among 120 patients with newly diagnosed functional area gliomas who underwent surgical treatment, 60 were included in each group: the integrated group with iMRI and fMRI and the conventional navigation group. Between-group comparisons were made for the extent of resection (EOR), preoperative and postoperative activities of daily living based on the Karnofsky performance status, surgery duration, and postoperative intracranial infection rate.

**Results:**

Compared to the conventional navigation group, the integrated navigation group with iMRI and fMRI exhibited significant improvements in tumor resection (complete resection rate: 85.0% vs. 60.0%, *P* = 0.006) and postoperative life self-care ability scores (Karnofsky score) (median ± interquartile range: 90 ± 25 vs. 80 ± 30, *P* = 0.013). Additionally, although the integrated navigation group with iMRI and fMRI required significantly longer surgeries than the conventional navigation group (mean ± standard deviation: 411.42 ± 126.4 min vs. 295.97 ± 96.48 min, *P*<0.0001), there was no significant between-group difference in the overall incidence of postoperative intracranial infection (16.7% vs. 18.3%, *P* = 0.624).

**Conclusion:**

The combination of 3.0 T iMRI with multimodal fMRI guidance enables effective tumor resection with minimal neurological damage.

## Background

Glioma is the most common primary malignant intracranial tumor, with an annual incidence of approximately 56 cases per 100,000 people worldwide [1]. Surgery remains the cornerstone of glioma treatment, with subsequent personalized treatment plans combining postoperative radiotherapy and chemotherapy [2]. Gliomas can affect sensorimotor, language, cognitive functional, and computational functional areas, the basal ganglia, internal capsule, thalamus, and distance sulcus in the cortex and subcortex. Damage to these brain functional areas can cause permanent neurological disorders, including postoperative paralysis, visual field defects, aphasia, and cognitive impairment [3].

The main goal of modern neurosurgical treatment for gliomas is achieving maximum safe tumor resection [4–6]. The extent of resection (EOR) is the only controllable clinical prognostic indicator during surgery [7–9]. With the introduction of new technologies in neurosurgery, standards have increased for maximum safe tumor resection. Specifically, maximum safe tumor resection of gliomas located in functional brain areas should not be sought at the expense of neurological function [10]. McGirt et al. reported that the 2-year survival rates in patients with glioblastoma without and with new intraoperative neurological deficits were 23% and 8%, respectively [11]. Therefore, surgery treatment of functional area gliomas should seek to achieve optimal EOR without causing new neurological deficits [12]. This study aimed to evaluate the clinical utility and safety of 3.0 T intraoperative magnetic resonance imaging (iMRI) combined with multimodality functional MRI (fMRI) guidance in the resection of functional area gliomas.

## Materials and methods

### Patient selection

In this retrospective cohort study, we analyzed the cases and imaging data of 538 patients who underwent glioma surgery in the Department of Neurosurgery at the First Affiliated Hospital of the University of Science and Technology of China (Anhui Provincial Hospital) between October 2020 and November 2022. We stratified the patients into two groups: the integrated navigation group with iMRI and fMRI and the conventional navigation group. Inclusion criteria for the integrated navigation group with iMRI and fMRI were as follows: 1) a primary diagnosis of gliomas in functional areas (lesions within or adjacent to the language, visual, or motor functional areas, as well as significant white matter fiber tracts);2) utilization of intraoperative electrophysiological monitoring; 3) postoperative pathological confirmation of glioma; 4) absence of any standard preoperative treatments such as radiotherapy or chemotherapy; 5) comprehensive preoperative and postoperative clinical data; and 6) informed consent provided. Exclusion criteria included: (1) patients undergoing reoperation for recurrent gliomas, (2) patients who only received biopsy surgery, and (3) patients with incomplete clinical data. 60 cases met the inclusion criteria for the integrated navigation group with iMRI and fMRI. Due to financial limitations, concerns over extended surgical and anesthesia times, and the inapplicability of high-field iMRI and multimodal fMRI for patients with internal metal implants, some individuals either declined or were ineligible for iMRI and multimodal fMRI scans, receiving surgery with conventional navigation instead. A neurosurgeon, using a one-to-one matching principle based on demographic and clinical characteristics such as gender, age, and tumor location, selected 60 patients to form the conventional navigation group. This study received approval from our institutional ethics committee and was conducted in compliance with the Declaration of Helsinki. Informed consent was obtained from all patients or their legal guardians.

We collected several sociodemographic and clinical variables: sex, age, preoperative symptoms, tumor location, preoperative (V1) and postoperative (V2) tumor volumes, preoperative and postoperative Karnofsky Performance Status (KPS) scores, duration of surgery, postoperative pathological stage, and incidence of postoperative intracranial infection. V1 and V2 were determined by two experienced neurosurgeons using validated imaging software, calculated as tumor volume = length × width × height × π/6. The extent of resection (EOR) was calculated using the formula EOR = (V1 − V2)/V1 × 100% and was categorized into four levels: gross total resection (100%), near-total resection (90–99%), subtotal resection (85–90%), and partial resection (< 85%) [12]. KPS scores were assessed through physical and neurological examinations one day before and seven days after surgery. Surgical duration was recorded from the operation reports. Cerebrospinal fluid analysis was performed on patients who exhibited high fever postoperatively, revealing abnormal cell counts and positive meningeal signs, leading to a diagnosis of postoperative intracranial infection.

### Preoperative imaging data and navigation plan

All patients in the integrated navigation group underwent preoperative head MRI, diffusion tensor imaging (DTI), fMRI, and magnetic resonance spectroscopy in the 3.0 T composite operating room (GE 3.0 T) of our hospital. Subsequently, a navigation plan was developed by transferring the DTI and fMRI data to the navigation workstation, followed by data preprocessing. The imaging data of each sequence were aligned into the same spatial coordinate system; additionally, the “object creation” module was used to delineate tumor range and generate a three-dimensional model of the lesion. Based on the preoperative characteristics and lesion severity, different scan sequences were used for different glioma grades (T1W-enhanced and T2/FLAIR sequences for high-grade and low-grade gliomas, respectively). The “BOLD mapping” and “fiber mapping” modules were used to reconstruct cortical functional areas and the pyramidal tract, respectively, to select a personalized surgical pathway. The conventional navigation group only underwent routine cranial MRI navigation sequences.

### Surgical procedure

**Fig. 1:**
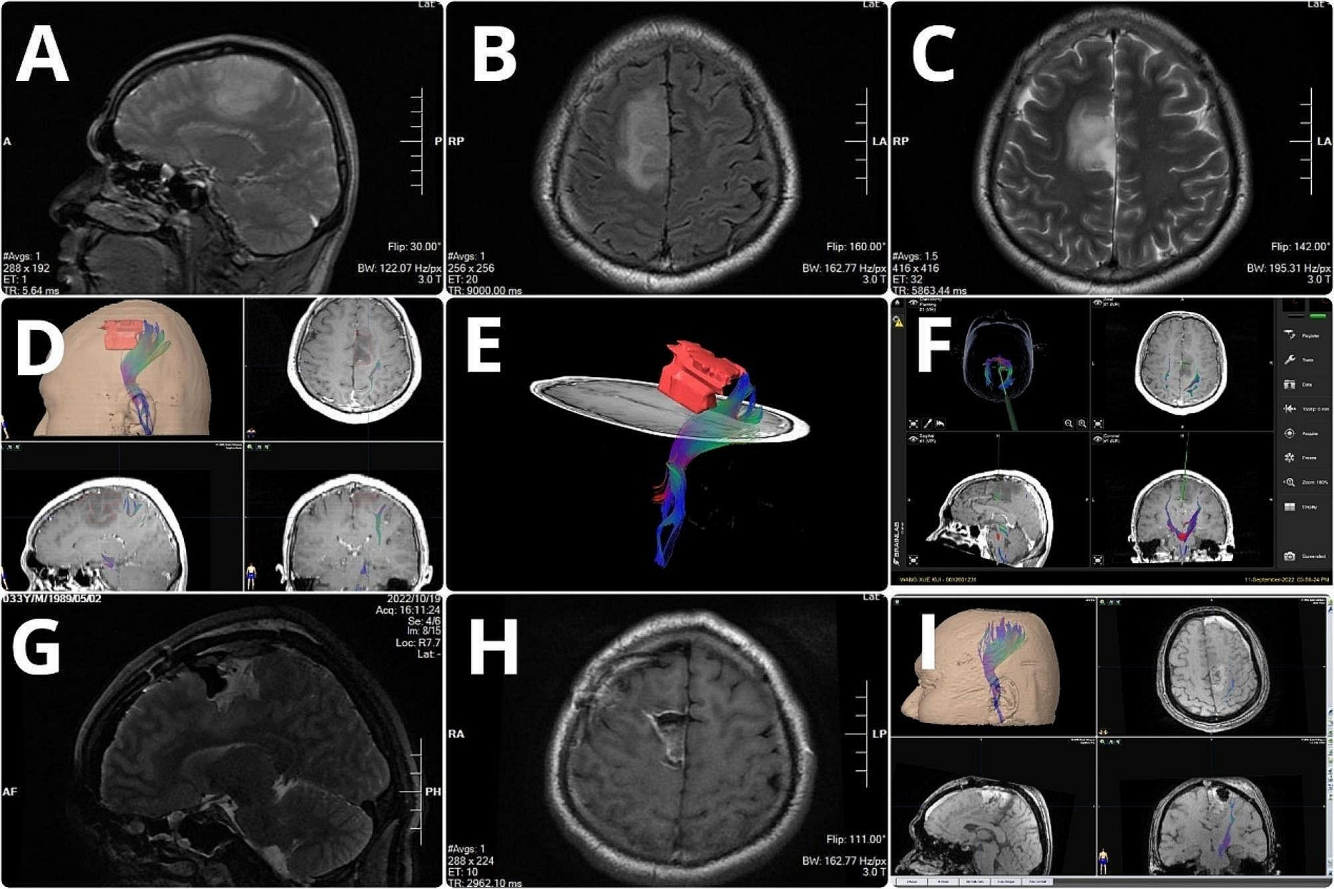
Case 1: A 33-year-old male patient. The preoperative MRI scan showed patchy long T1 and T2 signals in the right frontal lobe and subcortex (**A**–**C**). Preoperative three-dimensional reconstruction showing the spatial relationship between fiber tracts and tumor location (**D**, **E**). The preoperative plan was imported into the navigation system (**F**). Intraoperative MRI and intraoperative DTI three-dimensional reconstruction showed complete tumor resection and preservation of intact fiber tracts (**G**–**I**). DTI, diffusion tensor imaging; MRI, magnetic resonance imaging

**Fig. 2:**
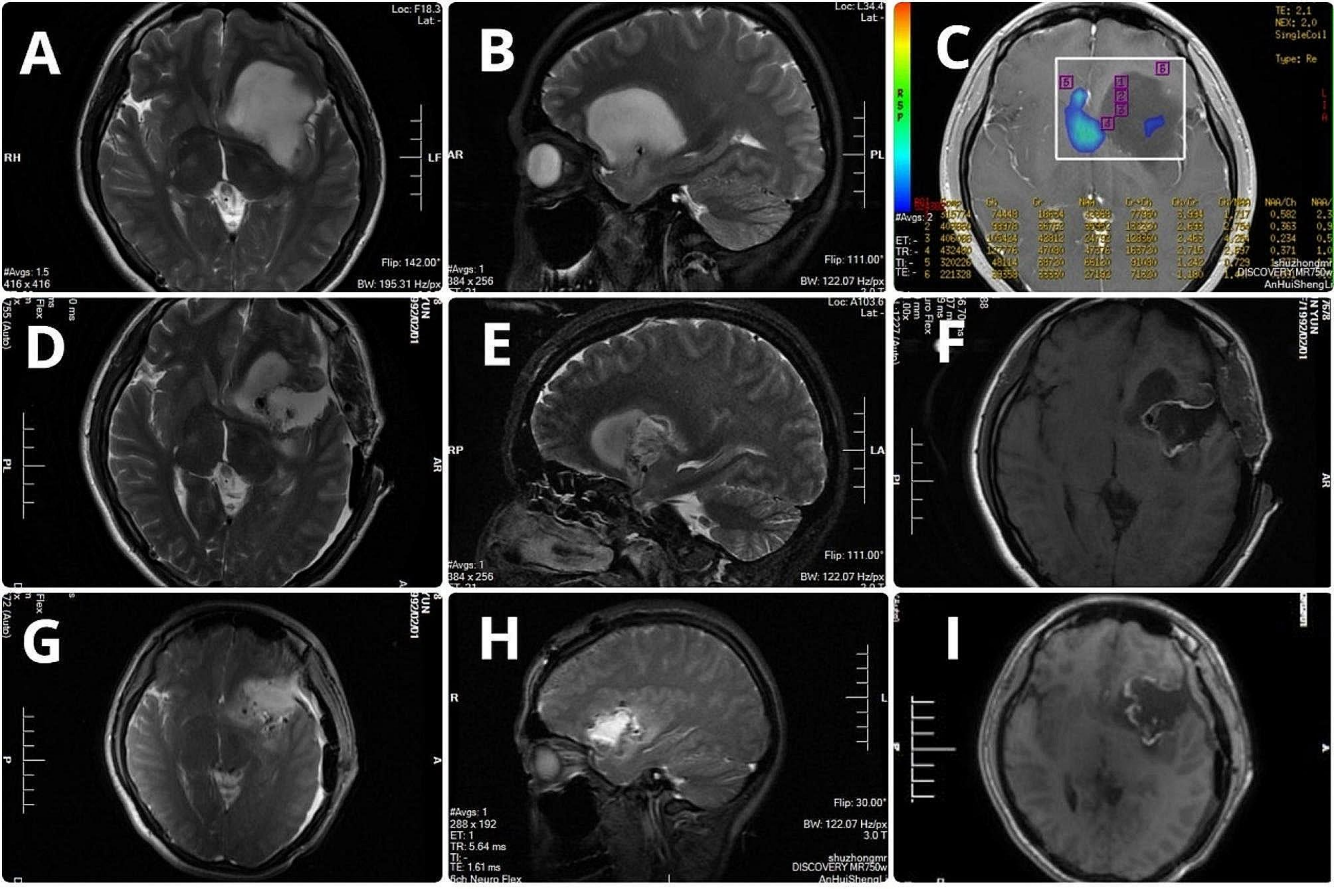
Case 2: Magnetic resonance imaging (MRI) of preoperative scan (**A–C**), first intraoperative MRI scan (**D–F**), and second intraoperative MRI scan. (**G–I**)

For preoperative navigation planning, three-dimensional reconstruction data of tumor and fiber tracts and cortical functional areas were imported into the neuronavigation system. Infrared matching was performed according to the patients’ facial features. After successful matching, the three-dimensional spatial positions of the lesion and adjacent fiber tracts were displayed on the screen. Intraoperatively, gradual navigation-guided tumor resection started at the farthest point from the functional area or fiber tracts(Fig. 1); subsequently, iMRI was performed to determine EOR in the following scenarios: (1) the surgeon subjectively determined that the tumor had been largely resected; (2) navigation indicated a < 5 mm distance between the cut margin and edge of the fiber tracts; (3) the surgeon could distinguish between the tumor and brain tissue edema under the naked eye and thus could not determine whether the tumor edge had been reached; (4) intraoperative severe brain displacement severely affected navigation accuracy; and (5) intraoperative electrophysiological monitoring showed that the motor or language area had been reached or neared. For intraoperative scanning, hemostasis was thoroughly applied to the tumor cavity; no hemostatic agents were used to ensure scanning quality. Subsequently, the intraoperative imaging data were transmitted to the navigation workstation and fused with the preoperative images. In case there was a residual tumor, the navigation plan was updated and the residual tumor was resected. This cycle could be repeated until iMRI confirmed complete or sufficient tumor resection(Fig. 2).

### Statistical analysis

Statistical analyses were performed using SPSS 26 (IBM SPSS Statistics 26). Normally and non-normally distributed data are expressed as the mean ± standard deviation and median, respectively. Between-group comparisons of normally and non-normally distributed data were performed using the t-test and U-test, respectively. Between-group comparisons of categorical data were performed the chi-square (χ^2^) test. Statistical significance was set at *p* < 0.05.

## Results

### Basic and clinical characteristics

The initial symptoms were headache and high intracranial pressure in 47 patients, epilepsy in 25 patients, muscle weakness in 26 patients, speech dysfunction in 9 patients, visual impairment in 3 patients, consciousness disorders in 1 patient, whereas 8 patients were asymptomatic. Postoperative pathology confirmed high- and low-grade gliomas in 90 and 30 patients, respectively. No adverse anesthesia reactions were observed in any of the patients during or after the surgery. There were no significant between-group differences in sex, age, tumor location, preoperative tumor volume, or preoperative Karnofsky scores (Table 1).

**Table 1:** General patient characteristics: between-group comparison

Characteristics	Integrated Navigation Group (*n* = 60)	Conventional Navigation Group (*n* = 60)	*P* -value
**Sex** (male/female)	35/25	35/25	1.000*
**Age** (mean ± SD, years)	51.90 ± 13.87	50.02 ± 13.89	0.459†
**Preoperative tumor volume** (mean ± SD, cm^3^)	51.24 ± 41.87	49.24 ± 39.48	0.791†
**Preoperative KPS score** (median ± IQR, score)	90 ± 10	90 ± 10	0.694‡
**Tumor location**			0.997*
Frontal lobe, corpus callosum	29	27	
Temporal lobe, insula	18	19	
Parietal lobe	7	8	
Thalamus, basal ganglia	5	5	
Brainstem	1	1	
**Tumor grade**			0.673^*^
LGG	16	14	
HGG	44	46	

* *P*-value obtained using Pearson’s chi-square test

^†^*P*-value obtained using independent two-sample t-test

^‡^*P*-value obtained using independent samples Mann–Whitney U test

Abbreviations: KPS, Karnofsky performance status; LGG, low-grade glioma; HGG, high-grade glioma; IQR, interquartile range

### Surgical efficacy and safety

Complete tumor resection rate was significantly higher in the integrated navigation group with iMRI and fMRI than in the conventional navigation group (Table 2). Ithe integrated navigation group with iMRI and fMRI, tumor infiltration in the functional area was present in three cases, complete resection could cause severe functional impairment. Therefore, only partial resection was achieved (EOR < 85%). Among partial resection cases, the minimum degree of tumor resection was significantly higher in the integrated navigation group with iMRI and fMRI than in the conventional navigation group (70.94% vs. 63.24%). These findings suggest that the integrated navigation group with iMRI and fMRI had a significantly increased complete resection rate and reduced degree of residual tumor. Notably, there was no significant between-group difference in preoperative KPS scores; in contrast, the postoperative KPS score was significantly higher in the integrated navigation group with iMRI and fMRI than in the conventional navigation group. Additionally, the postoperative change in KPS scores was significantly higher in the integrated than in the conventional navigation group. Surgery duration was significantly longer in the integrated than in the conventional navigation group. There was no significant between-group difference in the incidence of postoperative intracranial infection (Table 3).

**Table 2:** Between-group comparison of resection degree

Resection degree (EOR)	Total resection (%, cases)	Near-total resection (%, cases)	Subtotal resection (%, cases)	Residual (%, cases)
Integrated navigation group (*n* = 60)	85.0% (51)	6.7% (4)	1.7% (1)	6.6% (4)
Conventional navigation group (*n* = 60)	60.0% (36)	31.7% (19)	1.7% (1)	6.6% (4)

Degree of resection: Gross total resection (EOR = 100%), near-total resection (EOR = 90–99%), subtotal resection (EOR = 85–90%), and residual resection (EOR < 85%).

Statistical analysis was conducted using Pearson’s chi-square test.**χ**^**2**^ **= 12.37**,*P*** = 0.006**

**Table 3:** Between-group comparison of postoperative indicators

Observation indicators	Integrated navigation group (*n* = 60)	Conventional navigation group (*n* = 60)	*P*-value
Surgical duration (mean ± SD, min)	411.42 ± 126.4	295.97 ± 96.48	**0.001** ^*^
Postoperative infection rate	16.67% (10/60)	18.33% (11/60)	0.624†
Postoperative KPS (median ± IQR, points)	90 ± 25	80 ± 30	**0.013**‡
KPS difference value (median ± IQR, points)	0 ± 0	10 ± 20	**0.016**‡

^*^P-value obtained using Pearson’s chi-square test

^**†**^*P*-value obtained using independent two-sample t-test

‡ *P*-value obtained using independent samples Mann–Whitney U test

Abbreviations: KPS, Karnofsky performance status; IQR, Interquartile range

### Multifactorial analysis of factors influencing the postoperative KPS score

**Fig. 3:**
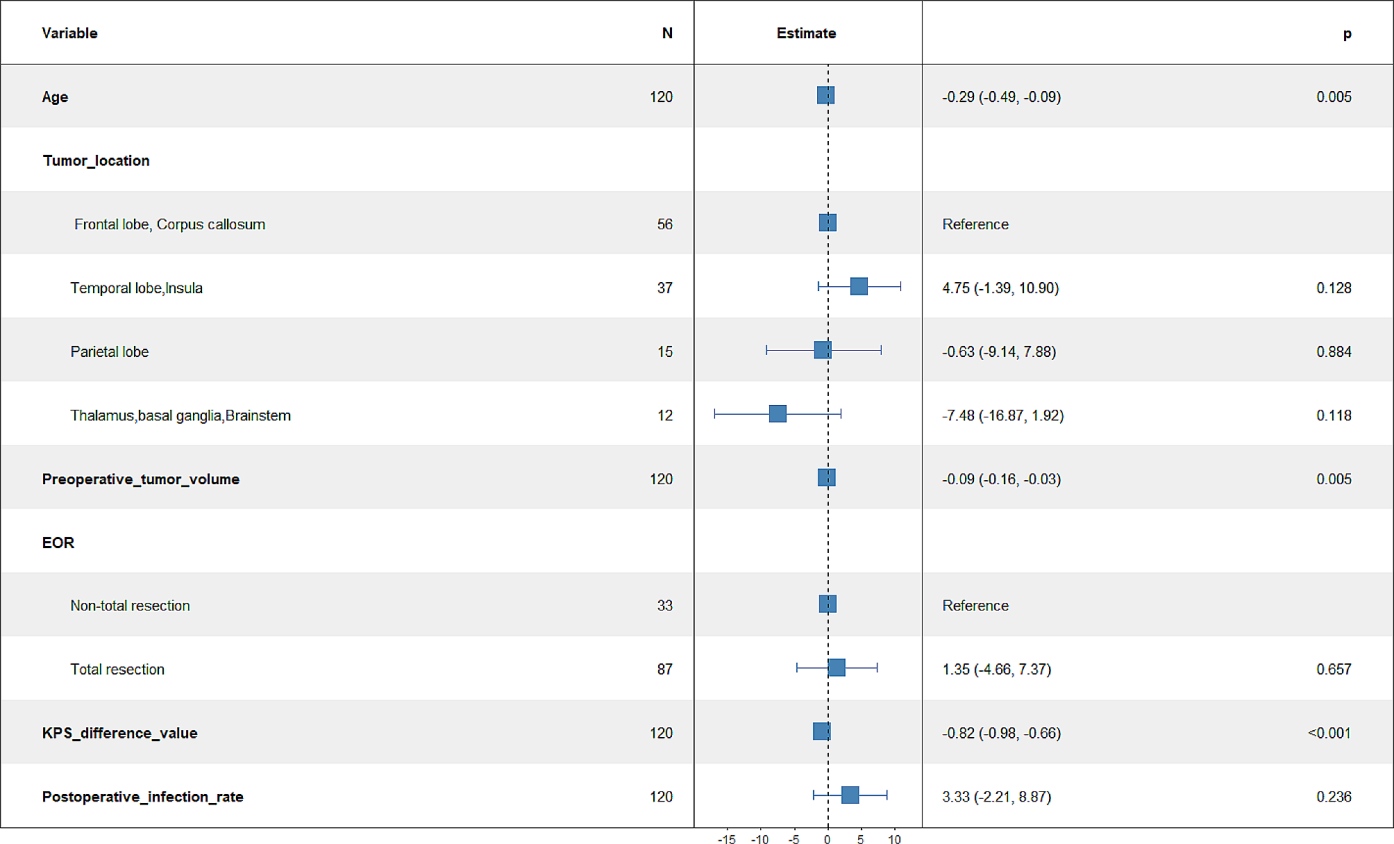
Multifactorial analysis of factors affecting postoperative KPS. EOR, Extent of resection; KPS, Karnofsky performance status

Multifactorial analysis revealed that age (odds ratio [OR]: −0.29, *P* = 0.005), tumor volume (OR: −0.09, *P* = 0.005), and postoperative KPS change (OR: −0.82, *P* < 0.001) negatively correlated with the postoperative KPS score (Fig. 3).

## Discussion

Surgery treatment of functional area gliomas should seek to achieve optimal tumor removal without damaging neurological function [13]. This necessitates accurate intraoperative identification of the spatial relationship between tumor lesion and cortical and subcortical fiber tracts to achieve precise tumor resection while preserving neurological function.

Awake craniotomy combined with direct electrical stimulation, used to map language, motor, and sensory functions during surgery, is considered the standard technique for resecting gliomas involving functional brain areas. It precisely identifies crucial neuronal structures within brain functional regions, thereby maximizing neurologic preservation while effectively enhancing the extent of glioma resection. This approach aims to improve patients’ progression-free survival and overall survival, ultimately enhancing postoperative quality of life [14]. However, practical challenges exist: ① Incorrect execution of stimulation methods, inappropriate parameter and intensity selection, incomplete exposure of functional areas, and other factors may lead to inaccurate identification of functional areas, resulting in false-negative or false-positive outcomes during surgery. ② Prolonged patient cooperation in awake conditions, compounded by discomfort such as pain, urinary catheter stimulation, and dry mouth, can induce fatigue and non-compliance, subjectively interfering with stimulation results. ③ Direct electrical stimulation during surgery may trigger seizures, prolonging surgical duration and increasing patient discomfort, sometimes necessitating temporary modifications to the surgical plan. ④ Awake craniotomy requires patients to be physically and mentally capable of undergoing the procedure, making it unsuitable for patients who refuse or are unable to follow instructions, experience respiratory difficulties, communication challenges, motor impairments, uncontrolled seizures, anxiety, obesity, or severe gastroesophageal reflux [15].

The emergence of new technologies has exposed several limitations of conventional neuronavigation during surgery for functional area gliomas; for example, loss of cerebrospinal fluid, gravitational effects, and surgery-induced brain displacement can result in navigation errors, which may lead to residual tumor or excessive resection [10, 16]. Accordingly, the limited ability of conventional neuronavigation to demonstrate spatial relationships between tumors and cortical and subcortical fiber bundles increases the risk of intraoperative neurological function damage. The aforementioned limitations can be mitigated using iMRI [17]. Moreover, fMRI and DTI can identify cortical and subcortical white matter fiber tracts as well as accurately outline three-dimensional spatial positions using digital post-image processing [18, 19]. Additionally, DTI can reveal tumor location and allow improved surgical guidance [20]. Accordingly, combining both techniques allows improved structural and functional visualization as well as safer and more accurate surgery approaches.

Studies have demonstrated the clinical utility of iMRI in glioma resection [6, 10, 13, 21]. However, the clinical utility and safety of intraoperative multimodal fMRI navigation, performed once or multiple times, still require further exploration in collaboration with colleagues. Our study yielded the following main findings. First, The rate of complete resection in the integrated navigation group with iMRI and fMRI showed a significant increase, with even the cases of incomplete resection demonstrating significantly lower residual tumor volumes compared to the conventional navigation group. Second, the postoperative KPS scores were significantly higher in the integrated navigation group with iMRI and fMRI compared to the conventional navigation group, indicating a significantly lower incidence of new postoperative neurological deficits in the former group.Third, duration of surgery was longer in the integrated navigation group due to factors such as iMRI scanning, intraoperative image processing and uploading, and re-disinfection; however, this approach allowed precise surgery with a relatively smaller surgical range. Nonetheless, the postoperative infection rate and the incidence of anesthesia-related complications in the integrated navigation group did not increase.

Our study showed a significantly higher infection rate in both groups, mainly due to our stringent criteria for infection. Patients who developed high fever postoperatively and tested positive for signs of meningeal irritation were considered suspected infection cases, and were subjected to biochemical, routine, and culture examination of cerebrospinal fluid. Even if the culture results were negative, patients with abnormal cerebrospinal fluid cell counts were categorized into the infection group. Furthermore, the high infection rate may also be attributed to the involvement of the tumor in the ventricles in some patients. These patients underwent ventricular opening during surgery (7 cases in the integrated navigation group with iMRI and fMRI and 8 cases in the conventional navigation group), and postoperatively presented with suspected signs of infection prompting cerebrospinal fluid examination, which revealed abnormal cell counts. However, bacterial cultures of the cerebrospinal fluid were negative. We included these patients in the infection group in our study, although they did not experience prolonged hospital stays or new complications due to factors such as fever.

Multimodal functional magnetic resonance imaging not only enables maximal safe tumor resection but also aids in the early recognition of tumor metabolism and molecular pathology. T2/fluid-attenuated inversion recovery mismatch signals can identify isocitrate dehydrogenase mutations; magnetic resonance spectroscopy can identify specific metabolites in the region of interest to distinguish tumor necrosis from solid tumor tissue, and can also identify the metabolic products produced by IDH-mutant tumors; diffusion-weighted imaging (DWI) using apparent diffusion coefficient (ADC) measurements from multiple diffusion gradients can predict tumor grading. DTI is more sensitive in depicting the affected parenchymal borders and can visualize white matter fiber tracts. With the advancement of molecular imaging, multimodal functional magnetic resonance imaging not only helps delineate tumor boundaries and visualize tissue structure more effectively, but also serves as a non-invasive method for identifying tumor grades, molecular features, and distinguishing tumor progression from pseudoprogression [22].

### Limitations

The sample size included in this study is small, with a particularly low proportion of low-grade glioma cases, which may have introduced some bias into the research results. In future studies, we will use a larger sample size, conduct multicenter research, and extend the follow-up period to further validate the advantages of intraoperative MRI combined with multimodal functional MRI navigation in functional area glioma surgeries.Various factors can result in inaccurate overlay of preoperative DTI and iMRI data, including loss of cerebrospinal fluid, gravity-induced brain displacement, and rebound of compressed fiber bundles after partial tumor resection. These may result in surgical inaccuracies, which may damage white matter fiber bundles during further tumor resection, resulting in new-onset neurological dysfunction. Although elastic fusion imaging technology has been proposed to address these concerns, [23] further studies are warranted to validate this issue and develop more convenient alternatives.

## Conclusion

Compared with the conventional navigation group, the integrated navigation group showed higher complete resection rate and lower incidence of new postoperative neurological function injuries. Although the integrated navigation group required a significantly longer time for surgery than the conventional navigation group, there was no significant between-group difference in the overall infection rate. Our findings indicate the safety and effectiveness of 3.0 T iMRI combined with multimodal fMRI navigation for the resection of functional area gliomas.

## Data Availability

No datasets were generated or analysed during the current study.

## Abbreviations

ADC: Apparent diffusion coefficient
DTI: Diffusion tensor imaging
DWI: Diffusion-weighted imaging
EOR: Extent of resection
fMRI: Functional magnetic resonance imaging
HGG: High-grade glioma
iMRI: Intraoperative magnetic resonance imaging
IQR: Interquartile range
KPS: Karnofsky performance status
LGG: Low-grade glioma
OR: Odds ratio
V1: Preoperative tumor volume
V2: Postoperative tumor volume
